# Supplementary material for: Heterogeneous recruitment abilities to RNA polymerases generate nonlinear scaling of gene expression with cell volume
Source: Nat Commun. 2021 Nov 25;12:6852. doi: 10.1038/s41467-021-26952-y (PMC8617254; doi:10.1038/s41467-021-26952-y)
Supplement: Supplementary file 3 — Reporting Summary [file 41467_2021_26952_MOESM3_ESM.pdf]

nature portfolio

Corresponding author(s): Jie Lin

Last updated by author(s): Aug 23, 2021

Reporting Summary

Nature Portfolio wishes to improve the reproducibility of the work that we publish. This form provides structure for consistency and transparency in reporting. For further information on Nature Portfolio policies, see our Editorial Policies and the Editorial Policy Checklist.

Statistics

For all statistical analyses, confirm that the following items are present in the figure legend, table legend, main text, or Methods section.

n/a	Confirmed	
x		The exact sample size (*n*) for each experimental group/condition, given as a discrete number and unit of measurement
x		A statement on whether measurements were taken from distinct samples or whether the same sample was measured repeatedly
	x	The statistical test(s) used AND whether they are one- or two-sided<br>*Only common tests should be described solely by name; describe more complex techniques in the Methods section.*
x		A description of all covariates tested
	x	A description of any assumptions or corrections, such as tests of normality and adjustment for multiple comparisons
	x	A full description of the statistical parameters including central tendency (e.g. means) or other basic estimates (e.g. regression coefficient) AND variation (e.g. standard deviation) or associated estimates of uncertainty (e.g. confidence intervals)
	x	For null hypothesis testing, the test statistic (e.g. *F*, *t*, *r*) with confidence intervals, effect sizes, degrees of freedom and *P* value noted<br>*Give P values as exact values whenever suitable.*
x		For Bayesian analysis, information on the choice of priors and Markov chain Monte Carlo settings
x		For hierarchical and complex designs, identification of the appropriate level for tests and full reporting of outcomes
	x	Estimates of effect sizes (e.g. Cohen's *d*, Pearson's *r*), indicating how they were calculated

Our web collection on statistics for biologists contains articles on many of the points above.

Software and code

Policy information about availability of computer code

Data collection	For the acquisition of cell cycle regulator genes or RNA polymerase II genes, we used the on-line tool AmiGO (2.5.15).<br>For the acquisition of other data, no specific softwares used.
Data analysis	For mathematical simulations, we used MATLAB (R2020b and R2021a). Codes have been uploaded to the following link (https://github.com/QirunWang/MATLAB-code-for-simulation).<br>For motif searching, we used the on-line tools CentriMo of MEME suite (5.3.2).<br>For GSEA, we used the package clusterProfiler (3.12.0) in R (3.6.1). Codes have been uploaded to the following link (https://github.com/QirunWang/R-codes-for-GSEA).<br>For functional enrichment analysis, we used the on-line tools Metascape (3.5)

For manuscripts utilizing custom algorithms or software that are central to the research but not yet described in published literature, software must be made available to editors and reviewers. We strongly encourage code deposition in a community repository (e.g. GitHub). See the Nature Portfolio guidelines for submitting code & software for further information.

Data

Policy information about availability of data

All manuscripts must include a data availability statement. This statement should provide the following information, where applicable:

- Accession codes, unique identifiers, or web links for publicly available datasets
- A description of any restrictions on data availability
- For clinical datasets or third party data, please ensure that the statement adheres to our policy

RNA-seq data from Yuping Chen, et.al. 2020 was deposited at GEO (GSE145206) (link https://www.ncbi.nlm.nih.gov/geo/query/acc.cgi?acc=GSE145206).

Promoter sequences of the yeast genome were obtained from Yeastract database (link http://www.yeastract.com/formseqretrieval.php).
The KEGG BRITE hierarchy file for budding yeast was obtained from KEGG database (link https://www.kegg.jp/kegg/brite.html).
Cell-cycle regulator genes and RNA polymerase II genes were obtained from Gene Ontology (GO) database (link http://geneontology.org/).

Field-specific reporting

Please select the one below that is the best fit for your research. If you are not sure, read the appropriate sections before making your selection.

[x] Life sciences [ ] Behavioural & social sciences [ ] Ecological, evolutionary & environmental sciences

For a reference copy of the document with all sections, see nature.com/documents/nr-reporting-summary-flat.pdf

Life sciences study design

All studies must disclose on these points even when the disclosure is negative.

Sample size	Not applicable. In our study, we didn't draw samples to reflect the general situation of the population. All statistical analysis, both the mathematical simulations and data analysis, were done in population level.
Data exclusions	Not applicable. We didn't exclude any data in our analysis.
Replication	Not applicable. We didn't perform any kinds of biological experiments.
Randomization	Not applicable. We didn't perform any kinds of biological experiments.
Blinding	Not applicable. We didn't perform any kinds of biological experiments.

Reporting for specific materials, systems and methods

We require information from authors about some types of materials, experimental systems and methods used in many studies. Here, indicate whether each material, system or method listed is relevant to your study. If you are not sure if a list item applies to your research, read the appropriate section before selecting a response.

Materials & experimental systems

n/a	Involved in the study	
[x]	[ ]	Antibodies
[x]	[ ]	Eukaryotic cell lines
[x]	[ ]	Palaeontology and archaeology
[x]	[ ]	Animals and other organisms
[x]	[ ]	Human research participants
[x]	[ ]	Clinical data
[x]	[ ]	Dual use research of concern

Methods

n/a	Involved in the study	
[x]	[ ]	ChIP-seq
[x]	[ ]	Flow cytometry
[x]	[ ]	MRI-based neuroimaging
